# Gut Pharmacomicrobiomics: the tip of an iceberg of complex interactions between drugs and gut-associated microbes

**DOI:** 10.1186/1757-4749-4-16

**Published:** 2012-11-30

**Authors:** Rama Saad, Mariam R Rizkallah, Ramy K Aziz

**Affiliations:** 1 The Egyptian Bioinformatics and Systems Biology Network (EgyBio.net), Cairo, Egypt; 2 The American University in Cairo, New Cairo, Egypt; 3 Department of Microbiology and Immunology, Faculty of Pharmacy, Cairo University, Cairo, Egypt; 4 Current address: Systems Biology Research Group, UC San Diego, 9500 Gilman Drive, La Jolla, CA, 92093-0412, USA

**Keywords:** Human microbiome project, Xenobitoics, Liver enzymes, Metagenome, Microbiota, Metabolomics, Metabonomics, Pharmacokinetics, Pharmacodynamics, Pharmacomicrobiomics

## Abstract

The influence of resident gut microbes on xenobiotic metabolism has been investigated at different levels throughout the past five decades. However, with the advance in sequencing and pyrotagging technologies, addressing the influence of microbes on xenobiotics had to evolve from assessing direct metabolic effects on toxins and botanicals by conventional culture-based techniques to elucidating the role of community composition on drugs metabolic profiles through DNA sequence-based phylogeny and metagenomics. Following the completion of the Human Genome Project, the rapid, substantial growth of the Human Microbiome Project (HMP) opens new horizons for studying how microbiome compositional and functional variations affect drug action, fate, and toxicity (pharmacomicrobiomics), notably in the human gut. The HMP continues to characterize the microbial communities associated with the human gut, determine whether there is a common gut microbiome profile shared among healthy humans, and investigate the effect of its alterations on health. Here, we offer a glimpse into the known effects of the gut microbiota on xenobiotic metabolism, with emphasis on cases where microbiome variations lead to different therapeutic outcomes. We discuss a few examples representing how the microbiome interacts with human metabolic enzymes in the liver and intestine. In addition, we attempt to envisage a roadmap for the future implications of the HMP on therapeutics and personalized medicine.

## Introduction

The gut microbiota is the most predominant and most diverse microbial community residing in the human body [1]. It comprises hundreds of microbial species, together constituting about 10 times the number of body cells [2,3], and contributes substantially to human metabolic processes to the extent that up to 36 % of small molecules in human blood are contributed by the gut microbiome [4]. The gut microbiota’s impact on drug response and metabolism has been explored since the mid 20^th^ century (reviewed in [5]); however, past studies have mostly focused on assessing the metabolic activity of gut microbial communities on antibiotics and botanicals [6-9]. Meanwhile, the influence of the host genetic makeup on drug response occupied the center stage of personalized medicine research, specifically in the clinical domain, leading to the rise of pharmacogenomic approaches to personalized therapy, while a pivotal player in xenobiotic metabolism, the microbiota, was mostly being overlooked [10,11].

The various metabolic capabilities of the gut microbiota fueled the study of its effects on drug metabolism [11,12]. Several approaches were adopted, including comparisons between metabolic patterns of conventional and germfree mice, biochemical assays of microbial metabolic activities in cultures, and mutagenicity tests [5,6,13]. Population-based approaches, such as investigating the correlation between compositional variations in gut microbiota and response to a particular drug, e.g., digoxin, were followed as well [9].

The evolution of microbial genomics from culture-based (i.e., sequencing genomes of bacterial species after isolating their colonies) to culture-independent strategies (metagenomics—or shotgun sequencing of microbial and viral communities [14,15]) has allowed the identification of the molecular signature of the gut microbiome associated with a certain disease or with altered drug response [16]. To describe this new expansion of pharmacogenomics, we suggested the term pharmacomicrobiomics to denote the effect of microbiome variations on drug disposition and response [17,18]; here, we apply this concept explicitly to the human gut microbiome, the best-studied microbiome for its effect on xenobiotics.

**Figure 1:**
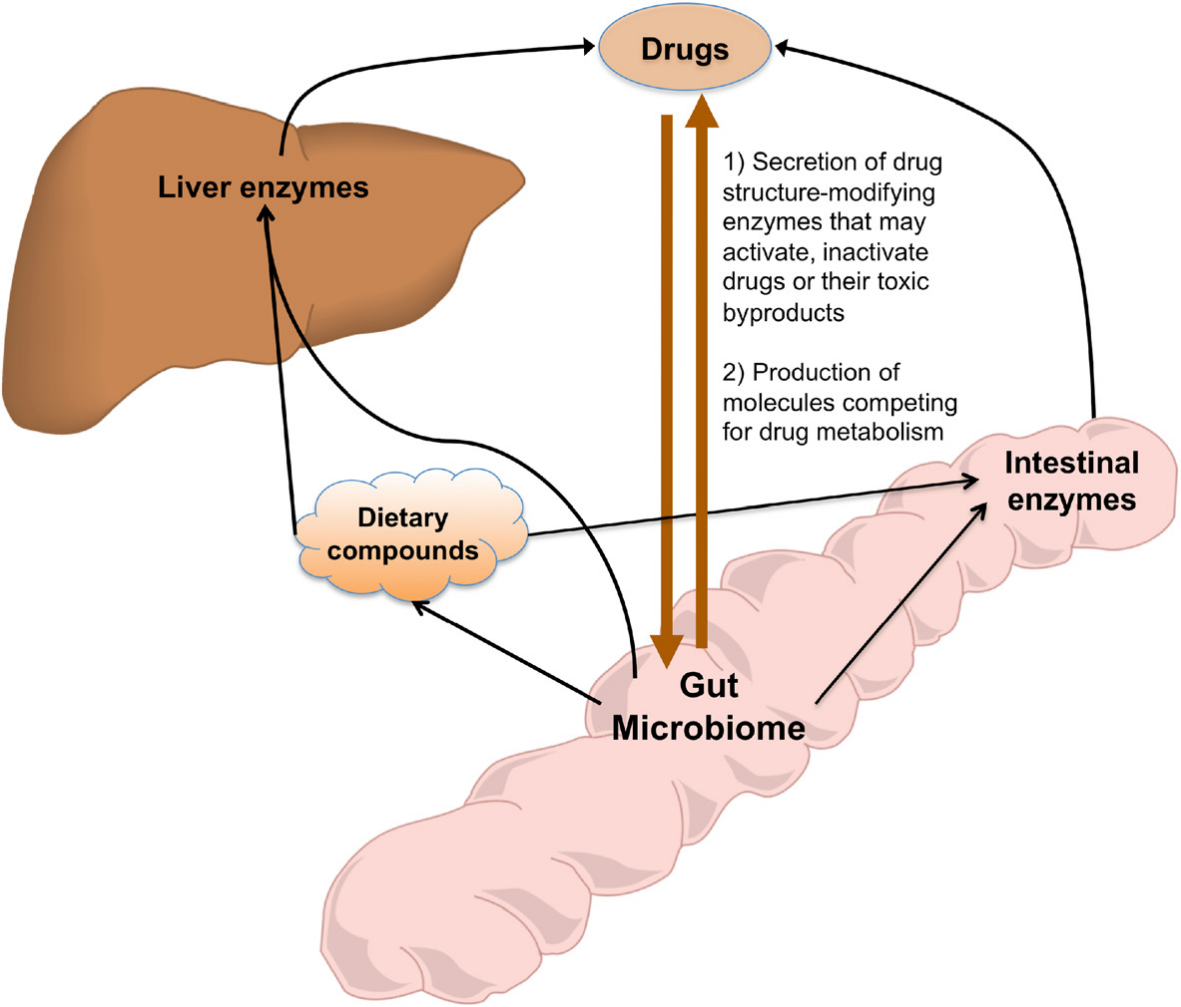
**Different ways of interactions between the gut microbiome and drugs**, **sometimes involving dietary compounds or intestinal and liver enzymes.** Liver and intestine cartoons were taken from the publicly available clipart of the University of Wisconsin, Madison. URL: http://www.biochem.wisc.edu/medialab/clipart.aspx..

In a broad sense, the term gut pharmacomicrobiomics encompasses the effect of the gut microbiome variations on pharmacokinetic and pharmacodynamic processes [17,19] (See Section “Term disambiguation”). However, to date, the better-documented effects of the human gut metagenome on drugs are those related to metabolism (i.e., effects on pharmacokinetic), either through: (i) the secretion of enzymes that modify the chemical structure of drug molecules, (ii) the secretion of metabolic products that interfere with drug metabolism, (iii) the modification of the levels and activities of liver and intestinal enzymes, or (iv) the modulation of expression of human metabolic genes [16] (Figure 1). Taking into consideration the enormous number of gut-associated microbes, and the extremely large number of diverse genes they encode and pathways they express, understanding the effect of the gut microbiota on human response to drugs is an indispensable step towards providing a comprehensively tailored/personalized therapy that would be more efficient, cost-effective, and with lower adverse drug events [17,20].

In this review, we aim at providing an overview of the influence of gut microbiota on drugs, spanning the documented metabolic effects of the microbiota and the different approaches used for their investigation. In addition, we provide an outlook for the future of pharmacomicrobiomics in the context of the Human Microbiome Project (HMP) and for the application of metagenomic approaches as an integral part of pharmacotherapy and personalized medicine.

### Term disambiguation

***•Microbiome and microbiomics***: To the best of our knowledge, the term ‘microbiome’ was first suggested in 2000 by the Nobel Laureate, Joshua Lederberg, to describe the sum of microbial genomes associated with the human body, which he described as a part of “the human extended genome” (URL: http://www.project-syndicate.org/commentary/microbiology-s-world-wide-web). Soon after, it was used in the same meaning in literature [21,22]. Currently, however, *microbiome* is being used to denote two different concepts: (i) the collective microbial genome of a community (i.e., microbial metagenome) or (ii) the sum of all microscopic life forms, viz. microbes, within an environment (i.e., micro.biome). *Microbiome* was initially confined to host-associated metagenomes, but is now being used interchangeably with microbial metagenome (e.g., the Earth Microbiome Project http://www.earthmicrobiome.org/[23]). The less frequently used term, ‘microbiomics’, describes the study of functional aspects related to the microbiome, including the integration of high-throughput genome-wide data [24].

***•Pharmacogenomics and pharmacomicrobiomics***: Pharmacogenomics [25] is a well-established term that describes the effect of human genome variations on drug disposition and action. The term can certainly be applied not just to the human nuclear and mitochondrial genomes, but also to the human extended genome or the genome of the human supraorganism [18]; yet, to specify the impact of the human-associated microbiome on drugs, we have coined the term pharmacomicrobiomics [17,18], which we consider as a natural expansion of pharmacogenomics, which is likely to spread when more HMP data accrue.

***•Metabolomics and metabonomics***: Those two verbally similar terms have been sometimes used interchangeably to describe the high-throughput study of all genome-encoded metabolites produced by a particular organism or a community; however, Nicholson and coworkers carefully denote the difference between the two terms as they use *metabolomics* to describe the study of genetically controlled metabolites and fluxes produced by one type of cells or tissues, whereas they define *metabonomics* as the measurement of metabolites produced by a collection of cells/genomes within a multicellular organism or an ecosystem [26] (the latter once described as the ‘meta-metabolome’ [27]).

### Role of gut microbiota in xenobiotic metabolism

The influence of the gut microbiota on the metabolism of xenobiotics has been regarded extensively as a response-modifying process, and several mechanisms have been proposed and demonstrated [11]. Gut-associated microbes can alter drug metabolism directly by producing enzymes that degrade or activate the drug molecules, or by competing with drug molecules over the metabolizing enzymes [17,20]. In addition, the gut microbiota may exert its influence by modulating the activity or altering the levels of the host’s drug-metabolizing enzymes or by producing enzyme-inducing metabolites that are originally derived from diet [28,29] (Figure 1). Accordingly, the microbiome’s response-modifying effect has been widely appreciated in nutrition and toxicology, and the role of gut microbiota in metabolism has initially been investigated in terms of the metabolism of compounds of dietary and botanical origin (Table 1).

**Table 1:** Role of gut microbiota in the metabolism of dietary compounds and phytochemicals

**Chemical** (**drug or herbal remedy**) {**CID**}	**Pharmacological effect**	**Role of gut microbiota in metabolism**	**Altered metabolism and subsequent outcome**	**References**
Heterocyclic aromatic amines (HAAs)	Carcinogenic agents	HAAs, originally derived from cooking proteins, are pro-mutagenic compounds known to be carcinogenic to rats and mice reviewed in [30]. Normally upon ingestion of a cooked protein, gut microbiota metabolize these compounds to yield unconjugated mutagen metabolites detectable in urine and stool, and human liver enzymes CYP450 IA1 and IA2 activate these compounds to the active mutagenic forms.	Enhancement of CYP450 activity, deconjugation of HAAs and consequent increased mutagenic activity	[29]
		The effect of elevated active mutagens metabolites was reported to be significantly higher in conventional rats than germfree rats. Conventional rats have shown elevated activity of ethoxyresorufin-O-deethylase (EROD), which is a CYP450-dependent enzyme responsible for the biotransformation of HAAs and is increased in the small intestine upon ingestion of fried meat. Thus, the intestinal microbiota is thought to play a central role in HAA metabolism and thereby, in the response to mutagens through enhancing the activity of CYP450 enzymes responsible for the activation of mutagens.		
Cycasin {5459896}	Toxic glycoside	Members of the gut microbiota hydrolyze cycasin into the carcinogenic derivative, methylazoxymethanol.	Microbiome-induced hydrolysis leading to direct toxic effect	[7]
Rutin {5280805}	A quercetin glucoside with angio-protective effects	Several gut anaerobes, e.g., *Bacteriodes uniformans*, *Bacteroides ovatus*, and *Butrivibrio* sp. hydrolyze dietary rutin into its corresponding quercetin aglycone and polyphenols. The release of both the free quercetin aglycone and the phenolic metabolites underlies rutin’s mutagenic effect and the further inhibition of platelet aggregation, respectively. The free quercetin aglycone is a mutagen. Furthermore, the administration of rutin has been correlated with the increase of mutagenic activity of other glycosides with mutagenic aglycone component. The increase in glycosidic activity was expected to further increase the release of quercetin; however, the activation of quercetin was decreased in rats fed with rutin in contrast to the free aglycones of other mutagens such as 2-amino-3-methylimidazo [4,5-f] quinoline (IQ), 2-amino-3,4-dimethylimidazo [4,5-f] quinoline (MeIQ), and 2-amino-3,8-dimethylimidazo-[4,5-f] quinoxaline (MeIQx).	Microbiome-induced hydrolysis leading to indirect mutagenic effect	[31]
Aflatoxin B1 {186907}	Carcinogenic mycotoxins	Rats with conventional gut microbiota have shown two-fold increase in aflatoxin concentration in S9 liver fraction. Additionally, an in vivo-modified Ames test showed that rats with conventional gut microbiota have higher number of mutants of the indicator organism, *Salmonella* Typhimurium TA98, than germfree rats.	Potentiated toxic effects	[31]
(+)- catechin and (−)-epichatechins {9064, 72276}	Anti-oxidants	The effects of (+)-catechins and (−)-epicatechins on liver and intestinal enzymes have been reported to be different between germfree rats and rats with human gut microbiota. In germfree rats, (+)-catechins and (−)-epicatechins resulted in increase in the levels of liver CYP450 2C11 and (+)- catechins caused elevation in the specific activity of liver Uridine 5'-diphospho-glucuronosyltransferase UGT-chloramphenicol. On the other hand, cytosolic glutathion-S-transferase (GST) levels were higher in rats harboring human gut microbiota upon the administration of (+)-catechins. However, in both germfree and human microbiota inoculated rats, (+)-catechins and (−)-epicatechins increased the specific activity of UGT-4-methyl umbelliferone in the intestine. Furthermore, the specific activity of intestinal UGT-chloramphenicol was higher in rats inoculated with human microbiota.	Indirect potentiating/lowering effect on drug metabolism depending on the type of co-administered drug, the metabolic pathway adapted, and the effect of the resulting metabolite	[32]
2-methoxy esterone	Anti-angiogenic	Members of the gut microbiota are believed to convert 2-methoxy esterone to the active steroid form. This was demonstrated upon incubation of 2-methoxy esterone with isolated rat cecum, where two different reactions were found to take place: oxidoreduction at C17 and demethylation at C2 resulting into the active form.	Oxidoreduction and demethylation resulting in activation of prodrug	[33]
Chlorogenic acid {1794427}	Antioxidant	Gut microbiota metabolize chlorogenic acid to 3-hydroxycinnamic acid and 3-(3-hydroxyphenyl)propionic acid, which are subjects to phase II conjugation followed by excretion in urine. In absence of gut microbiota, chlorogenic acid is metabolized to benzoic acid, which in turn is conjugated with glycine yielding hippuric acid. Gonthier et al. found that the bioavailability of chlorogenic acid relies on its metabolism by gut microbiota [34].	Microbial metabolism resulting in potentiated clinical effect	[34,35]
Soy-derived phytoestrogens	Xeno-estrogens	Some microbial communities in the gut produce active metabolites from soy-derived phytoestrogens resulting in enhanced efficacy. In addition, the phytoestrogens metabolites produced by gut microbiota are suggested to affect cytochrome P enzymes, which are responsible for estrogen hydroxylation, and therefore result in lower toxic events.	According to the type of microbiota present, toxicity or lower action may result.	[36,37]
Baicalin {64982}	Potential antioxidant, anti-inflammatory and liver tonic	Gut microbiota normally hydrolyze baicalin into its corresponding aglycone baicalein, which is readily absorbable and subject to re-conjugation following absorption. Absence of gut microbiota in germfree rats reportedly resulted in lower levels of baicalin in plasma following oral administration.	Potentiated clinical effect	[38]
Anthocyanins {145858}	Potential anticancer, anti-oxidant and anti-inflammatory	Gut microbes are responsible for the hydrolysis of the glycosidic linkage between the sugar and the aglycone by means of β-glucosidases resulting in the release of the free aglycone active form.	Microbial hydrolysis leading to activation of prodrug	[39]
Genistin {5281377}	Anti-cancer, estrogenic and antiatherosclerotic	Gut microbes hydrolyze the glycosidic linkage between the sugar and the aglycone by means of β-glucosidases resulting in the release of the free aglycone active form genistein.	Microbial hydrolysis leading to activation of prodrug	[39]
Naringin {442428	Anti-oxidant, anti-cancer and blood cholesterol lowering effect	Same as with anthocyanins and genistin, microbial β-glucosidases lead to the release of the free aglycone active form naringenin.	Microbial hydrolysis leading to activation of prodrug	[39]

*CID* = Chemical ID from the PubChem database (URL: http://pubchem.ncbi.nlm.nih.gov) [40] is provided in curly braces for all drugs or botanicals.

Additionally, the absence of conventional gut microbiota in germfree mice has been correlated with perturbations in levels of liver and intestinal metabolic enzymes in comparison to their corresponding levels in mice with conventional gut ecosystem [29], and conventional gut microbiotas in human and mice were shown to be associated with a modest elevation in the levels of drug-metabolizing enzymes, such as sulfotransferase1 B1 (SULT1B1) and with reduced levels of other enzymes, such as SULT1C1, NAT1 and NAT2 [28] (Table 2).

**Table 2:** **Effect of microbiota on hepatic and intestinal metabolic enzymes **[28]

**Enzyme**	**Function**	**Effect of gut microbiome**
Ethoxyresorufin-O-deethylase (EROD)	A CYP450-dependent enzyme responsible for the biotransformation of HAAs	The presence of normal gut microbiota in rats potentiates EROD activity upon ingestion of fried meat
Glutathione S-transferase A 1/2 (GSTA1/2)	Being among the alpha class of GST enzyme family that is preferentially expressed in the colon rather than the liver, it plays a central role in phase II detoxification of xenobiotics. In addition, GSTA1/2 class displays a glutathione peroxidase activity, which underlies its antioxidant and cyto-protective effects.	Measuring GSTA1/2 levels in both germfree rats and microbiota–reassociated rats showed 4- and 5-fold increase in the enzyme level in germfree males and females, respectively.
Glutathione S-transferase A4(GSTA4)	Among the alpha class of GST enzymes that possess high affinity to alk-2-enes	Germfree rats showed 1.5- and 1.9-fold increase in the levels of GSTA4 than microbiota–reassociated rats in males and females, respectively.
Glutathione S-transferase M1 (GSTM1)	GSTM1 is one of the mu class of GSTs which detoxify carcinogens, toxins, drugs and oxidative stress products.	Germfree female rats showed a statistically significant but modest elevation in colonic GSTM1 levels compared to rats with gut microbiota. However, male rats didn't exhibit this elevation. This increase in germfree female rats may be coincidental in spite of the statistical significance.
Epoxide hydroxylase 1 (EPHX1) enzyme	Responsible for the activation and detoxification of xenobiotics as polycyclic aromatic hydrocarbons	Germfree rats showed a substantial increase in the colonic levels of EPHX1 than rats associated with rat gut microbiota.
Epoxide hydroxylase 2 (EPHX2) enzyme	Located in cell cytosol and perixosomes and detoxifies specific peroxides by catalyzing their conversion into dihydrodiols	Germfree rats showed a moderate increase in the colonic levels of EPHX2 than rats associated with rat gut microbiota.
Sulfotransferase 1C2 (SULT1C2) enzyme	Among the SULT1 enzyme subfamily, which conjugates phenolic compounds with sulfo groups obtained from 3'-Phosphoadenosine-5'-phosphosulfate (PAPS)	Germfree female rats showed a statistically significant modest increase (1.6-fold) in colonic levels of SULT1C2.
Sulfotransferase 1B1 (SULT1B1) enzyme	A member of the SULT1 enzyme subfamily	On the contrary to SULT1C2, germfree male and female rats showed a statistically significant decrease (0.4- and 0.6-fold, respectively) in the enzyme level than gut microbiota- associated rats.
N-acetyltransferase 1 (NAT1) & N-acetyltransferase 2 (NAT2) enzyme	Detoxify hydrazine and arylamine drugs	NAT enzyme levels were modestly elevated in germfree rats in comparison with rats with conventional gut microbiota.
Glutathione peroxidase 2 (GPX2) enzyme	A selenium-dependent member of the GPX family of glutathione peroxidase that is present in the epithelium of the gastrointestinal tract	Elevated GPX2 mRNA levels have been correlated with the reintroduction of microbiota in germfree rats.

### Impact of microbiome variations on drug response and toxicity

Most studies on drug-microbe interactions did not take in consideration the microbiome profile/composition of an individual or a population; however, these variations are the basis of pharmacomicrobiomics, and their study has become possible now that the HMP has been established [3,41], and HMP data have already been made available [1,42].

Several studies associated a particular 16S rRNA microbial signature with specific biomarker metabolites and clinical outcomes. This association has been extended to encompass several conventional drugs such as digoxin and acetaminophen [9,43]. Profiling the signatures of the microbial communities in relation to their metabolic effect on drugs among patients is likely to introduce clinical markers that will dictate treatment regimens tailored in accordance with each patient's resident microbiota [43]. Such regimens, in turn, will modify the current treatment strategies that are based on conventional pathologic and pharmacokinetic parameters to take into account the interindividual perturbations in the gut microbiota and the gut ecosystem. These measures are especially true with the evidence of the sym-xenobiotic metabolism that involves both the host and the associated microbiota to biotransform drugs, including first-line therapies [9,44] (Table 3). As a consequence, microbiome-labile medications may be limited, or their dose readjusted, for certain populations or individuals harboring particular gut microbial community profiles. Several drugs that possess structural similarity to microbial products and are thereby potential candidates of microbial metabolism are yet to be studied.

**Table 3:** **Role of gut microbiota in the metabolism of conventional first line therapies and over**-**the**-**counter **(**OTC**) **drugs**

**Drug** {**CID**}	**Pharmacological effect**	**Role of gut microbiota in metabolism**	**Effect of microbiota on clinical outcome**	**References**
Acetaminophen {1983}	Analgesic and antipyretic	Competitive o-sulfonation between p-cresol, produced by some gut bacterial communities, and acetaminophen increases acetaminophen toxicity. Therefore, assessment of microbiome activity has been suggested as a guideline prior to the administration of acetamniophen.	Exaggerate clinical effect and toxicity	[43]
Chloramphenicol {5959}	Antibiotic	Some patients display bone marrow aplasia following the oral administration of chloramphenicol owing to the presence of coliforms that mediate the metabolic conversion of chloramphenicol to a toxic form known as p-aminophenyl-2-amin-1,2-propanediol.	Increase toxicity	[45]
Digoxin {2724385}	Cardiotonic	Altered concentration of *Eggerthella lenta* between populations affects the concentration of reduced digoxin metabolite. 36 % of North Americans vs. 13.7 % southern Indians were able to metabolize digoxin, a difference that was correlated with altered concentrations of *E*. *lenta* between the two populations. Concomitant administration of digoxin and erythromycin or tetracycline resulted in digoxin intoxication. Accordingly, it is recommended to avoid the concurrent use of both medications.	Potentiate both activity and toxicity	[8,9]
Flucytosine {3366}	Antifungal	Patients who have received antibiotics showed lowered metabolic transformation of flucytosine (commonly known as 5-fluorocytosine) to 5-fluorouracil (5-FU).	Potentiate effect	[44]
Metronidazole {4173}	Antibiotic: antifungal and antimicrobial (against anaerobic microbes)	*Bacteroides fragilis* is among gut commensals, and its infection is commonly treated by metronidazole. A strain of *B*. *fragilis* that overexpresses *recA* was resistant to metronidazole in comparison to the wild-type strain. Inactivation of *recA* resulted in the increased sensitivity to metronidazole, and the *B*. *fragilis recA* mutants had more double strand breaks.	Provide resistance to the antimcrobial/antifungal effect	[46]
Metronidazole {4173}	Antibiotic: antifungal and antimicrobial (against anaerobic microbes)	Comparison of metronidazole metabolites between germfree rats and conventional rats showed the exclusive excretion of the metabolites by conventional rats. Those metabolites were retrieved upon adding *Clostridium perfringens* to metronidazole.	Lower the effect by activating metabolism	[13]
Sulfasalazine	Azodyes/Antibiotics	Salfasalazine is a prodrug that requires activation by azoreduction, mediated by intestinal bacteria, to result in sulfapyridine and 5-aminosalisylic acid. Patients who have undergone ileostomy had lower plasma levels of sulfapyridine than controls. Futhermore, antibiotic administration resulted in decrease of the azoreduction split. Intestinal microbiota mediate the clearance of both sulfapyridine and 5-aminosalisylic acid, where the decrease in acetylation rate is associated by increased side effects.	Activate the drug	[47]
Sulfinpyrazone {5342}	Azodyes/Antibiotics	The gut microbiota plays a major role in the azoreduction of sulfinpyrazone. Ilesotomy patients had dramatically lower levels of the sulfide form than controls (the area under the curve, AUC, for sulfide metabolite was 25-fold lower in the plasma in case of ileostomy patients).	Activate the drug	[47]
Sulindac {1548887}	Non steroidal anti-inflammatory drug (NSAID)	Sulindac is a prodrug that undergoes reductive metabolism by gut microbiota and liver enzymes into an active sulfone metabolite. Patients with ileostomy exhibited half the AUC following 12 hours of oral administration of 200 mg dose.	Activate the drug	[47,48]
Sorivudine {5282192}	Antiviral	A toxic interaction was reported in 18 Japanese people upon concomitant oral administration of sorivudine and 5-FU. *Bacteroides* sp. are responsible for this toxicity owing to their production to (E)-5-(2-bromovinyl) uracil (BVU) metabolite which in turn deactivates dihydropyrimidine dehydrogenase (DPD) responsible for the metabolism of 5-FU. Germfree rats had significantly lower BVU levels in both urine and blood.	Increase toxicity	[49,50]
Zonisamide {5734}	Anticonvulsant	Gut microbiota is central to the metabolism of zonisamide by reduction producing 2-sulfomoyacetylphenol. Germfree rats had lower levels of this metabolite, and its levels were increased after those rats were inoculated with gut microbiota.	Lower the effect	[51]

CID = Chemical ID from the PubChem database (URL: http://pubchem.ncbi.nlm.nih.gov) [40] is provided in curly braces for all drugs.

### A systems biology view of the host-microbiota metabolome and co-metabolome

Previously reported drug-microbe and drug-microbiome interactions have mostly been described as phenotypic observations of drugs being modified by a microbial species, an entire microbial community, or an even more intricate system consisting of a microbial and a human component. However, in many cases the process, biochemical pathway, or specific reaction remains unknown, which renders the analysis of those interactions by reductionist approaches difficult. Instead, exploring the causality of those interactions might require systems approaches such as the metagenomic analysis of the microbial community followed by the identification of differentially abundant or differentially expressed candidate genes or genomic subsystems [52] involved in those interactions. Yet, metagenomic surveys that determine microbial community profiles, gene presence/absence and abundance, or functional classification of sequence fragments are not sufficient to tell a coherent story about the observed phenotypes since a gene’s presence does not imply its expression or functionality. Consequently, extracting knowledge from those microbiome explorations and translating them into an ultimately tailored therapy requires modeling the human microbiome, variome, and interactions between them via integrating multiple layers of information, including transcriptomic, proteomic, and metabolomic data. Such integration is not always achievable in a system with this complexity. For instance, a statistically sound correlation between mRNA and protein expression levels in mid-log phase *Saccharomyces cerevisiae* cells has been hindered by technical limitations [53]. If this was the case with a unicellular organism or with relatively uniform cell lines [54], then further levels of complexity are to be expected in the gut microbiome ecosystem, where communities of unicellular organisms coexist in balance with the human multicellular tissues. Systems biology approaches for such complex communities are inevitable but are still in early development [55,56].

From a holistic perspective, tailoring a pharmacotherapy that accommodates intraindividual and interindividual variations would take into account the variations in the host’s genetic makeup, its associated-microbiome, and metabolomic interactions between the host and its associated microbiota (i.e., co-metabolome). With the recognition of the considerable role of the human microbiome and its variations together with the formerly well-recognized role of the human variome in predicting response to pharmacotherapy, there is a growing demand in both clinical and research domains for proper computational models that are able to comprehensively consider all such aspects of variability [26,53,54]. The best-recognized process in altered drug response, controlled by both human genome and microbiome, is the presystemic metabolism or first-pass effect (reviewed in [57]). Since the metabolism of xenobiotics in humas is performed by host and microbial enzymes, the metabolic process is recognized as combinatorial or “sym-xenobiotic” as recently described [10]. Furthermore, a continuous metabolic interaction, termed metabolome-metabolome interaction, exists between the host and its associated microbiota [10]. Modeling the human/microbiome variations and metabolome-metabolome interactions will provide insights into the metabolism of xenobiotics and thereby allow for accurate predictions for drug response [26].

Nicholson and colleagues [26] attempted to visualize the role of both the host and its associated microbiota in xenobiotic metabolism in the gut by proposing an interesting model, assuming six different cell types in both host and microbiota, every type of which has its own transcriptome and metabolome depending on its role. There is a mutual metabolic exchange between the host and microbiota, and the extracellular compartment contains metabolites generated by both of them. Those metabolites are the result of drug and food metabolism, and might lead to metabolic alterations in both the host and its associated microbiota. This probabilistic model of metabolism was introduced in an attempt to tackle the potential interaction between the different host- and microbiome-related factors that would eventually display a certain outcome for metabolism. The model likens the complex process of drug metabolism to a Japanese Pachinko (pinball machine), where pins represent enzymes and transporters involved in metabolism, holes indicate outlets for metabolites, and pathways are represented by the sequence of pins. According to this model, the final outcome is the increment of the probabilities of collisions between pins and balls [26].

### Web resources for exploring gut pharmacomicrobiomics

**•Human variome resources**:

•HVP (Human Variome Project): http://www.humanvariomeproject.org[58]

•HapMap: http://hapmap.ncbi.nlm.nih.gov[59]

**•Human microbiome resources**:

•MetaHIT (Metagenomics of the Human Intestinal Tract): http://www.metahit.eu[42]

•HMP: http://hmpdacc.org[41]

**•Tools or databases for browsing the human microbiome**:

•IMG/HMP: http://www.hmpdacc-resources.org/cgi-bin/imgm_hmp/main.cgi[41]

•myMGDB: http://edwards.sdsu.edu/cgi-bin/mymgdb/show.cgi

•MG-RAST: http://metagenomics.anl.gov[60]

•The SEED Servers: http://www.theseed.org/servers[61]

**•Pharmacogenomics**/**pharmacomicrobiomics databases**:

•PharmGKB (Pharmacogenomics Knowledge Base): http://www.pharmgkb.org[62]

•PacDB (Pharmacogenetics and Cell Database): http://www.pacdb.org[63]

•CTDB (Comparative Toxigenomics Database): http://ctdbase.org[64]

•The PharmacoMicrobiomics Portal: http://www.pharmacomicrobiomics.org[65]

**•Enzymes**/**pathways databases**:

•KEGG (Kyoto Encyclopedia of Genes and Genomes): http://www.genome.jp/kegg/[66]

•Model SEED: http://seed-viewer.theseed.org/seedviewer.cgi?page=ModelView[67]

•BRENDA (BRaunschweig ENzyme Database): http://www.brenda-enzymes.org[68]

### Future anticipations

**Figure 2:**
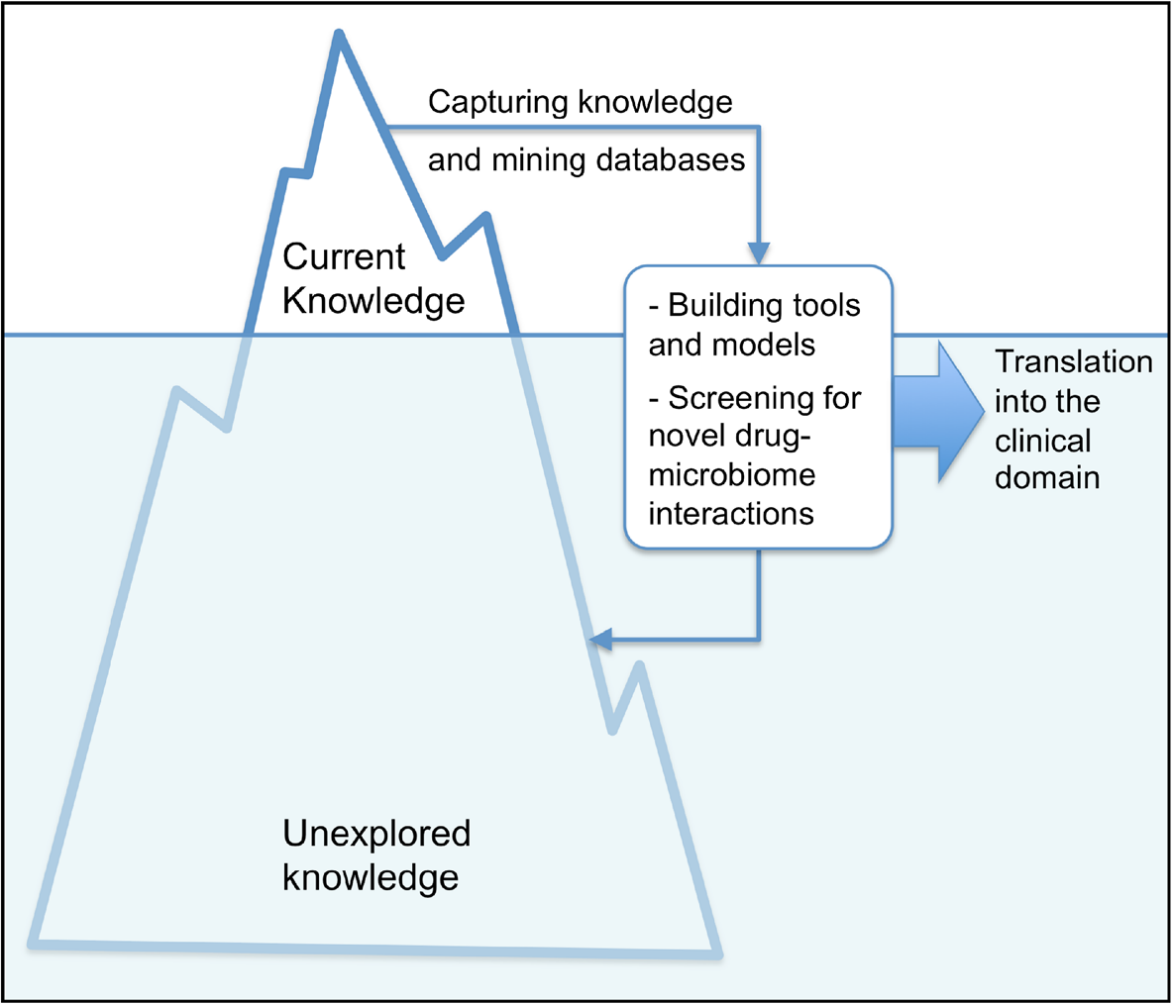
A roadmap for the development of the nascent field of gut pharmacomicrobiomics and its translation into the clinical domain.

The current advances in the Human Variome Project [69,70] and the HMP [3,41], together with a battery of publicly available web resources (See Section “Web resources for exploring gut pharmacomicrobiomics”) offer a starting point for those interested in drug-microbiome interactions to address several intriguing questions. However, the examples reported previously (e.g., those in Tables 1, 2 and 3) are just the tip of an iceberg of yet-to-be-discovered interactions between the host variome, associated microbiome, their combined metabolome, and chemicals ingested by humans. Ultimately, the study of those interactions in spite of their complexity is driven by the need for devising personalized therapeutic regimens aiming at optimizing drug bioavailability to obtain maximal efficiency and minimal toxicity. Below, we suggest a roadmap of four steps for the development of the nascent field of gut pharmacomicrobiomics and its translation into personalized medicine (Figure 2).

#### Capturing current knowledge

The first step is to capture and organize the currently available information on drug-microbiome complex interactions by building databases similar to those built for pharmacogenetics, pharmacogenomics, and drug-drug interactions. Concomitant with building databases is developing tools and resources to support discovery by mining those databases and connecting them to microbial genomic databases (e.g., SEED [61], GOLD [71]) metagenomic/microbiome databases, (e.g., MG-RAST [60], myMGDB, HMP [41], METAHIT [42]), and metabolic pathway databases (e.g., KEGG [66], modelSEED [67], BRENDA [68]).

#### Developing and performing high-throughput screens for novel drug-microbiome interactions

In parallel with capturing existing knowledge, there is continuous need for digging deeper into the unknown drug-microbiome interaction space. Addressing this need can be achieved via studies involving high-throughput screens of drugs against human microbiota from different individuals looking at the overall action of these microbiotas on representatives of different drug classes, or, reciprocally, via screening individual resident gut microbes against large libraries of drugs or other chemicals.

#### Developing software and building models for drug response simulation

The accumulated data in literature pointing out to the response variation mediated by mammalian host variome and microbiome calls for the construction of modeling software that considers all such parameters to provide rational hypotheses or accurate predictions for research [26,55,72]. Developing such modeling software and using it in building models requires encoding data compiled from the literature regarding the host variome, microbiome and co-metabolome, and incorporating these encoded data into a model capable of retrieving an informative index describing the predicted outcome. For instance, Hlavaty and colleagues [73] used a similar approach to construct a predictive model of an apoptotic pharmacogenetic index for infliximab in treatment of Crohn disease. Following data mining, they used SAS® enterprise miner software to analyze all the genetic variants involved with the apoptotic response of infliximab, they managed to develop a new pharmacogenetic index ranging from 0 which denotes diminished response to 3, indicating a powerful response [73].

#### Data integration and translation into the clinical domain

The availability of web resources, the generation of more data, and the construction of rigorous models for drug-microbiome interactions will offer a great opportunity to translate this knowledge into diagnostic and clinical measures. In the future, routing clinical practices should include integrating microbiome data and processing them to produce valid assumptions of clinical outcome, based on which the type, dose, and regimen of treatment will be planned for each patient. Accordingly, each case will have its own panel of personalized therapy. For instance, patients harboring gut microbiota known to be associated with elevated levels of metabolic enzymes will be scheduled for higher doses; patients with higher susceptibility to acetaminophen toxicity might either be given a lower dose or an alternative nonsteroidal anti-inflammatory medicine; and patients with a microbiota with higher ability to metabolize digoxin will be scheduled to receive lower dose.

## Conclusions

Throughout the past five decades, the study of the effect of gut microbiota went through several phases uncovering its ample significance in drug response. With the continuous growth of the HMP and its expansion to cover diverse human populations, it is anticipated that the primary data concerning the common gut microbiome profile and its diversity among humans will be revealed, enabling to pursue further studies on its effect on drug response among populations. However, several steps are yet to be taken in anticipation of the floods of HMP data, including the construction of databases, software, and models that would provide credible predictions of differential clinical outcome and fuel further hypothesis-driven studies whose findings might be integrated into clinical settings.

## Competing interests

The authors declare that they have no personal or financial competing interests.

## Authors’ contributions

RS reviewed literature, collected data, outlined and drafted the manuscript, and participated in writing the final version. MRR collected data and abstracts, and participated in writing the final version. RKA conceived the article, reviewed literature, and wrote the article in its final format. All authors read and approved the final manuscript.

## Authors’ information

Rama Saad is currently a visiting scholar at Vanderbilt University Medical Center, Nashville, TN, USA.
